# Adherence to Prudent and Mediterranean Dietary Patterns Is Inversely Associated with Lung Cancer in Moderate But Not Heavy Male Polish Smokers: A Case-Control Study

**DOI:** 10.3390/nu12123788

**Published:** 2020-12-10

**Authors:** Iwona Hawrysz, Lidia Wadolowska, Malgorzata Anna Slowinska, Anna Czerwinska, Janusz Jacek Golota

**Affiliations:** 1 Department of Human Nutrition, University of Warmia and Mazury in Olsztyn, Sloneczna 45f, 10-718 Olsztyn, Poland; malgorzata.slowinska@uwm.edu.pl; 2 Independent Public Complex of Tuberculosis and Lung Diseases in Olsztyn, 10-357 Olsztyn, Poland; aczerwinska@pulmonologia.olsztyn.pl; 3 Clinic of Thoracic Surgery, Medical Center Ars Medica, 10-513 Olsztyn, Poland; januszgolota@vp.pl

**Keywords:** lung cancer, men, smoking, smokers, dietary patterns, prudent diet, Mediterranean diet, diet quality score

## Abstract

Lung cancer is the most commonly diagnosed cancer in men worldwide. Studies regarding dietary patterns (DPs) and lung cancer are limited, with results remaining inconclusive, and the association of DPs with lung cancer in smokers is unclear. This study analyzed the associations between DPs, including the Polish-adapted Mediterranean diet (Polish-aMED) score, and lung cancer risk in Polish adult male smokers. This case-control study involved 439 men aged 45–80 years from northeastern Poland, including 187 newly diagnosed lung cancer cases. Dietary data was collected with a 62-item food frequency questionnaire (FFQ-6). Two approaches were applied to identify dietary patterns. The Polish-aMED score was calculated (hypothesis-driven approach) and a principal component analysis (PCA) was used to identify PCA-driven DPs (data-driven approach). A logistic regression analysis was performed to estimate the odds ratio (OR) and 95% confidence interval (95% CI) of the lung cancer risk associated with the adherence to DPs overall as well as for moderate (2.5–11 pack-years) and heavy (>11 pack-years) smokers. Among moderate smokers, the risk of lung cancer was lower by 41% (OR: 0.59; 95% CI: 0.39–0.90; *p* < 0.05; adjusted model) in the higher adherence to the prudent DP when compared to the lower adherence, and by 66% (OR: 0.34; 95% CI: 0.15–0.76; *p* < 0.05; adjusted model) in the high adherence (7–9 points) to the Polish-aMED score when compared to the low adherence (0–3 points). No significant association between the westernized traditional DP or the sweet dairy DP and lung cancer was revealed. In conclusion, the current study suggests that pro-healthy dietary patterns, including the Mediterranean pattern, may favour lower risk of lung cancer in moderate smokers, although it was not confirmed in heavy smokers.

## 1. Introduction

There were an estimated 18 million cancer cases around the world in 2018; of these, 9.5 million cases were in men and 8.5 million in women [1]. Cancer is the second most common cause of death globally (cardiovascular diseases were the most common) [2]. In 2018, GLOBOCAN [1] estimated that the most common cancers in the world were lung cancer in men and breast cancer in women, each representing 12.3% of the total number of new cases diagnosed. Lung cancer has become the most common cause of cancer death in men aged 40 and older and women aged 60 and older [3].

In Poland, the number of cases of malignant cancer in the last three decades has grown more than twice, in 2013 reaching over 156 thousand cases (equally 78 thousand in men and women). Lung cancer is the most commonly diagnosed cancer and the most common cancer cause of death in men in Poland and across the world. Lung cancer accounts for approximately 18.7% and 30.7% of the total number of cancer cases and deaths in men in Poland, respectively [4]. In Poland, longevity for men over the past few decades has been lower than in more developed European countries. This is partly due to the higher likelihood of unhealthy lifestyle behaviours of Polish men, e.g., tobacco use, alcohol abuse, and an unhealthy diet, resulting in more deaths from heart disease, cancer, and other diseases [5]. Cancer aetiology is multi-factorial, and includes some predictors that cannot be modified, such as age, genetic predisposition, and some environmental factors (including air pollution), whereas lifestyle factors, such as smoking, physical activity, and diet are modifiable and can be changed [2,3,6,7,8]. 

Tobacco smoking remains the predominant risk factor for lung cancer development [3]. It is estimated that about 90% of lung cancer cases worldwide are attributable to tobacco use (including passive smoking) [3,7]. Smoking generates thousands of free radical particles, and is one of the key sources of inflammation and oxidative stress, which can be neutralized by a dietary intake of antioxidants and/or anti-inflammatory foods, leading to a protective effect on lung function [9]. The consumption of fruits and vegetables with a high content of antioxidant vitamins, phenolic compounds, minerals, and dietary fiber has a beneficial effect on respiratory health [10,11]. Incidence rates of lung cancer vary among world regions as a reflection of different historic patterns of tobacco exposure, which includes exposure intensity and duration, cigarette type and degree of inhalation, and the evolution of these patterns over time, commonly called the tobacco epidemic [12]. It should be noted that lung cancer in inactive smokers, i.e., passive smokers (25% of lung cancer cases worldwide), is a disease different from the more common forms of lung cancer associated with active smoking [12,13,14]. Because tobacco remains the leading risk factor for lung cancer, prevention of this disease is focused on smoking avoidance and cessation. Other prevention measures include healthy diet choices and maintaining a physically active lifestyle [3]. However, research on the association of dietary patterns and lung cancer risk that takes into consideration smoking is still limited. There are suggestions that diet may act independently or in concert with tobacco smoking in shaping the descriptive epidemiology of lung cancer [8,15]. 

According to the World Cancer Research Fund [7,16], there is strong evidence that taking high doses of beta-carotene supplements (in current and former smokers) increases the risk of lung cancer. Contrarily, there is some evidence that consuming vegetables and fruit (in current smokers and former smokers), foods containing retinol, beta-carotene or carotenoids, foods containing vitamin C (in current smokers), and foods rich in isoflavones (in never smokers) might decrease the risk of lung cancer [7,17,18]. Further evidence suggests that the consumption of red meat, processed meat, and alcoholic drinks might also increase the risk of lung cancer [7,19]. Evidence for the impact of other food groups or nutrients of the risk of lung cancer is limited, and to date, no explicit conclusions have yet been drawn [7]. 

It is important to identify dietary factors that might be useful in lung cancer prevention [7]. Many previous studies have investigated single foods or nutrients to assess the association with lung cancer [19,20,21,22,23,24,25,26]. Since different foods are consumed in combinations, and they interact with each other in a complex way, a comprehensive dietary pattern analysis can better reflect dietary habits and provide an evaluation of the overall effects of diet on human health [27,28].

The research in the world on the relationship between dietary patterns (DPs) and lung cancer, particularly the effects of the Mediterranean diet, is ambiguous and limited. Many studies have investigated the associations between dietary patterns and lung cancer risk [29,30,31,32,33,34,35,36,37]. Though the findings are not definitive, the majority of these studies suggest that a healthy diet is associated with a decreased risk of lung cancer. A healthy diet (also known as a prudent diet) is usually characterized by high consumption of vegetables, fruit, white meat, fish, and whole grains, and low consumption of red meat, high-fat foods, and refined grains [30,38].

The Mediterranean diet (MD) has long been recognized as the optimal diet responsible for longer life and reduced cancer risk [39,40,41,42,43], and a few studies have demonstrated a strong and inverse relationship between high adherence to the Mediterranean diet and lung cancer [35,44,45,46,47]. There are many variants of the MD; each Mediterranean country has its own gastronomy habits [48,49]. However, this dietary pattern in different countries is characterized by some common features, i.e., high consumption of whole grains, fruit, and vegetables, moderate consumption of dairy products, olive oil, poultry, and fish, and low consumption of red meat and sweets. A final characteristic of this diet is the moderate consumption of alcohol (mainly red wine during meals) [50,51,52]. The MD is rich among others in fiber, vitamins, phenolic compound (polyphenol), and PUFA n-3 [53,54,55]. 

The results of the meta-analysis and reviews confirm that high adherence to the prudent pattern and the Mediterranean diet reduces the risk of many cancers [39,55], including lung cancer [30,47]. Certainly, the effect is not due to single nutrients, but to a complex of components with synergistic and antagonistic interactions [47]. However, the evidence is still limited, because there are still only a few studies (including one study of Polish men) focusing on dietary patterns [29,30,31,32,33,34,35,36,37] and the Mediterranean diet [34,44,45,46,47] and the risk of lung cancer. To the authors’ knowledge, no studies have so far been published assessing the relationship between dietary patterns and the risk of lung cancer in Polish men who smoked moderately or heavily.

The current study analyzed the associations between dietary patterns, including Polish-adapted Mediterranean diet (Polish-aMED) score and lung cancer risk in Polish adult male smokers.

## 2. Materials and Methods 

### 2.1. Ethical Considerations

The study protocol was approved by the Bioethics Committee of the Faculty of Medical Sciences, University of Warmia and Mazury in Olsztyn on 2 October 2013 (resolution no. 29/2013). All of the subjects gave their written informed consent to participate in the study.

### 2.2. Study Design and Sample Characteristics

A case-control study was carried out from October 2013 to August 2017. The total sample screened consisted of 439 adult men aged 45 to 80 (mean = 62.6, SD = 7.2) years. The subjects were residents of northeastern Poland. 

**Figure 1:**
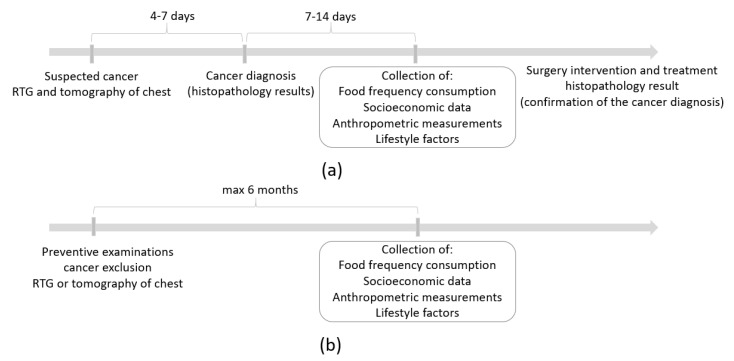
Time schemes of the study design for (**a**) the cancer sample and (**b**) the control sample. RTG digital X-ray examination.

The cancer sample consisted of 187 men with a newly primarily diagnosed (with digital X-ray examination (RTG) and computed tomography (CT) of the chest) and histologically confirmed lung cancer. The period from cancer diagnosis to case recruitment for study and data collection ranged from seven days to 14 days (Figure 1a). Cases during or after active treatment were not qualified for the study, since they may have involved changes in dietary habits or other behaviors. Therefore, the dietary data have not been disturbed by a possible change of diet after the diagnosis of cancer. The exclusion criteria of the cancer sample collection were described previously [34]. In brief, cases diagnosed with other cancer or secondary lung cancer, or with benign changes, after active treatment (e.g., chemotherapy, radiotherapy) or surgical intervention were not eligible for participation in the study.

**Figure 2:**
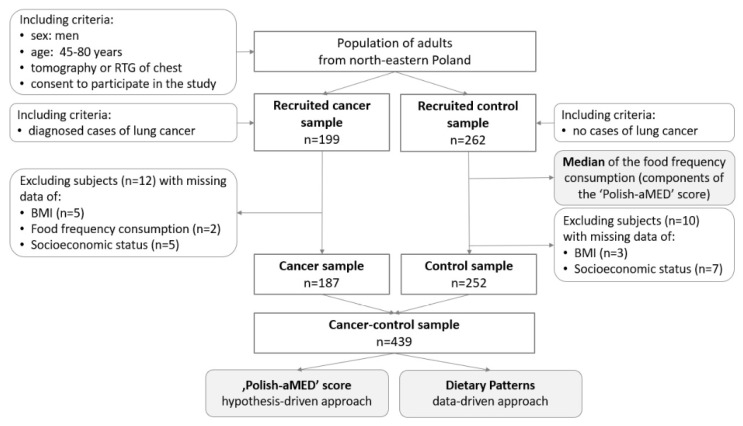
Flow chart of the sample collection and study design. Notes: RTG: digital X-ray examination; BMI: body mass index; Polish-aMED: Polish-adapted Mediterranean diet score.

The control sample consisted of 252 men who received a negative result from a digital X-ray examination (RTG) and/or computed tomography (CT) of the chest. The period since the cancer exclusion until participation in the study did not exceed six months (Figure 1b). Control subjects did not have any clinical symptoms or suspicion of any type of cancer in their medical history. The control sample was comprised of men recruited from those who attended national screening programs for the early diagnosis of lung cancer. Details regarding the enrollment are presented in Figure 2.

### 2.3. Dietary Data Collection and Dietary Pattern Identification

The dietary data collection methods have been previously described in detail [34]. Briefly, the subjects’ diets were studied using data from a validated food frequency questionnaire (FFQ-6) [56]. The FFQ-6 provided data regarding the consumption frequency of 62 food items. A qualified dietitian asked the participants during face-to-face interviews how frequently, on average, during the past year they had consumed the specific food items. The subjects could choose one of six categories (next converted into daily frequency): never or almost never (0 times/day), once a month or less (0.25 times/day), several times a month (0.1 times/day), several times a week (0.571 times/day), daily (1.0 time/day), or several times a day (2.0 times/day) [57]. 

Two approaches were applied to identify DPs. The Polish-adapted Mediterranean diet (Polish-aMED) score was calculated (hypothesis-driven approach), while the principal component analysis (PCA) was used to identify PCA-driven DPs (data-driven approach) [58].

The methods of deriving PCA-driven DPs have been previously described in detail [28,29]. After aggregating some food items collected with the FFQ-6, a total of 23 food groups were included in the PCA (Table S1). The data were checked using the Kaiser–Meyer–Olkin (KMO) index to measure the sampling adequacy [59], and Bartlett’s test of sphericity was used [60]. The PCA performance is justified when the KMO is greater than 0.5 and Bartlett’s test is statistically significant (*p* < 0.05). In this dataset, the Kaiser–Meyer–Olkin (KMO) value was 0.716 and Bartlett’s test had a significance of *p* < 0.001. For the statistical analysis, the sample size was sufficient for the PCA performance because the subject-to-item ratio was 19:1 (439 subjects and 23 items) [61]. Before the analysis, input variables were standardized. During the identification of the number of DPs, the following criteria were considered: (i) the eigenvalues of the variable correlations > 1.0, (ii) the plot of eigenvalues, and (iii) the total variance explained [58]. Rotated factor loadings with an absolute value > |0.30| were considered to be specific to the given pattern and used to label the patterns accordingly [62]. For each subject and each pattern, DP scores were calculated as a product of factor loading and food consumption frequency (for dietary variables). Next, for each DP, tertile intervals were calculated, which aimed to categorize subjects’ adherence to the patterns; subjects that were located in the upper tertile were characterized as those with higher adherence to the pattern, while subjects located in the bottom tertile were characterized as those with lower adherence.

The Polish-aMED score is a Polish version of the Mediterranean diet (MED) score, modified and described earlier by Fung et al. [63]. Briefly, the Polish-aMED score was calculated based on the qualitative data of the frequency of consumption (times/day) of nine selected dietary items. The methods of calculation of the Polish-aMED score have been previously described in detail [34]. In this study, alcohol was included as a component of the Polish-aMED score because there is no definitive evidence that it is a risk factor for lung cancer [7,64,65]. Detailed calculation of the Polish-aMED is shown in Supplementary Table S2. Possible scores for the Polish-aMED ranged from 0 (minimal adherence) to 9 (maximal adherence). Next, the adherence to the Polish-aMED was categorized as low (0–3 points), moderate (4–6 points), and high (7–9 points).

### 2.4. Smoking

Data regarding smoking status was collected for all subjects, including current smoking status, duration, the intensity of smoking, and time since cessation. The pack-years of smoking were calculated by multiplying the average number of packs of cigarettes smoked per day (intensity) by the number of years the person had smoked [66]. Smoking status pack-years were classified into two groups: moderate (2.5–11 pack-years) or heavy (>11 pack-years) smokers. These categories were based on the median value, which in this sample was equal to 11 pack-years (at enrollment).

Example of calculation:

A man reported smoking 30 cigarettes a day for 10 years.
The calculated number of pack-years is: 10 × 30/20 = 15 pack-years
10—years of smoking
30—number of cigarettes per day
20—number of cigarettes in one pack

### 2.5. Confounders

Data regarding socioeconomic, lifestyle, and medical factors were collected during an individual interview with each subject. The confounding factors were selected based on data analysis and a literature search. These factors included variables with a significantly differing distribution between the cancer and control samples, and the factors that were previously indicated as playing a role in lung cancer aetiology [7]: age, weight (body mass index), smoking, socioeconomic status, physical activity, the occurrence of lung cancer in a relative, workplace exposure to asbestos, chemical compounds (radon, uranium, chromium, arsenic, nickel, zinc, and polycyclic aromatic hydrocarbons), and hardwood dust. The potential confounders considered in this study have been described previously [33,34]. 

To describe socioeconomic status, the SES index was calculated using three variables: place of residence (three categories), education level (three categories), and economic status (three categories). The SES index was calculated as the sum of the values assigned to the individual response categories to each SES factor. All variables that were components of the SES index were standardized, and the tertiles of the SES index were then created to identify subjects with low, average, and high SES.

Subjects were asked about their physical activity at work and in leisure time. These data were combined, and three categories of overall physical activity were established (Table S3). Measurements of weight and height were taken, and body mass index (BMI, kg/m^2^) was calculated and used as an indirect measure of energy balance.

### 2.6. Statistical Analysis

The data are presented as percentages of the sample for categorical variables, or means and standard deviations (SDs) for continuous variables with normal distribution. Differences between groups were verified with Pearson’s chi-squared test for categorical variables or the Kruskal–Wallis test for continuous variables, and were considered statistically significant when *p* < 0.05. Logistic regression was used to verify the associations between DPs or Polish-aMED, lung cancer, and smoking. The odds ratios (ORs) and 95% confidence intervals (95% CIs) were calculated [67]. Two models were created and adjusted for potential confounders: age (categories), sex, BMI (categories), current smoking status (never smokers, current smokers, former smokers), socioeconomic status (low, average, high), overall physical activity (low, moderate, high), the occurrence of lung cancer in relatives (yes, no, I do not know), and occupational exposure in the workplace (yes, no) (see Section 2.5). The reference categories (OR = 1.00) were the control sample and the lowest level of each DP (the bottom tertile or low level). The level of significance of the OR was verified with Wald’s test [67]. The statistical analysis was performed using STATISTICA software (version 13.3 PL; StatSoft Inc., Tulsa, OK, USA; Kraków, StatSoft Polska). 

## 3. Results

The distribution of the general characteristics of the cases and controls is shown in Table 1. Most subjects with lung cancer cases were slightly older and had lower body weight compared to the non-cancer group (controls). When compared to the controls, more cases of lung cancer had a lower education level and socioeconomic status, and were less physically active at work and overall. A higher proportion of cases were former smokers who smoked for a long time (>10 years) and were classified as heavy smokers (>11 pack-years). The frequency of food consumption by smoking status is shown in Supplementary Table S4.

### 3.1. Food Consumption Frequency and Dietary Patterns

Using three PCA-driven DPs, three dietary patterns were derived: prudent DP, westernized traditional DP, and sweet dairy DP. The total explained variation of these three dietary patterns was 31%. Each dietary pattern explained 15%, 10%, and 6% of the consumption variance of 23 food groups, respectively. The concise names of the patterns represented the most prominent features and were labelled as follows: prudent DP—was positively loaded by the consumption frequency of whole grain products (0.65), fruits (0.64), nuts and seeds (0.57), vegetables (0.49), fish (0.48), legumes (0.47), and fruit, vegetable, and vegetable-fruit juices (0.32), and was negatively loaded by the consumption frequency of refined grain products (−0.54), sugar, honey and sweets (−0.45); westernized traditional DP—was positively loaded by the consumption frequency of red and processed meats (0.63), white meat (0.56), potatoes (0.51), other fats (0.45), vegetables (0.43), refined grain products (0.39), sweetened beverages and energy drinks (0.38), and sugar, honey, and sweets (0.37); sweet dairy—was positively loaded by the consumption frequency of animal fats (0.66), milk, fermented milk drinks, and curd cheese (0.49), sweetened milk drinks and flavored homogenized cheese (0.45), eggs (0.43), cheese (0.38), sugar, honey, and sweets (0.37), breakfast cereals (0.37), refined grain products (0.35), vegetable oils (0.33), and dried fruit and preserves (0.32), and was negatively loaded by the consumption frequency of other fats (−0.39) (Table 2). The frequency of food consumption by different dietary patterns is shown in Supplementary Table S5.

For the Polish-aMED, a significantly positive correlation was found between the Polish-aMED score with the consumption frequency of seven (out of nine) components: whole grains products (r = 0.49), fruits (r = 0.25), nuts and seeds (r = 0.54), vegetables (r = 0.50), legumes (r = 0.41), alcohol (r = 0.21), and the ratio of vegetable oils to animal fat (r = 0.11) (Table 2).

### 3.2. Dietary Patterns and Lung Cancer 

The association between dietary patterns and the risk of lung cancer are presented in Table 3 (distributions) and Table 4; Table 5 (odds ratios).

Within the cancer-control sample, one out of the three PCA-driven dietary patterns, prudent, showed a significant association with the prevalence of lung cancer in a logistic regression analysis. The risk of lung cancer was lower by 37% (OR: 0.63; 95% CI: 0.39–1.00; *p* < 0.05; crude model) in subjects with moderate adherence to the prudent DP when compared to the lower adherence as a reference. This association was not significant after adjustment. The risk of lung cancer was lower by 43% (OR: 0.57; 95% CI: 0.44–0.72; *p* < 0.0001; crude model) and 28% (OR: 0.72; 95% CI: 0.53–0.96; *p* < 0.05; adjusted model) in subjects with higher adherence to the prudent DP when compared to the lower adherence as a reference (Table 3). The westernized traditional DP and sweet dairy DP were not significantly associated with the risk of lung cancer. For the Polish a-MED DP, the risk of lung cancer was lower by 49% (OR: 0.51; 95% CI: 0.31–0.81; *p* < 0.05, adjusted model and OR: 0.51; 95% CI: 0.32–0.81; *p* < 0.05; adjusted model) in subjects with moderate adherence (4–6 points) and high adherence (7–9 points) to the Polish-aMED score when compared to the low adherence (0–3 points).

For moderate smokers, the risk of lung cancer was lower by 49% (OR: 0.51; 95% CI: 0.37–0.71; *p* < 0.0001; crude model) and 41% (OR: 0.59; 95% CI: 0.39–0.90; *p* < 0.05; adjusted model) for higher adherence to the prudent DP when compared to the lower adherence. The westernized traditional DP and sweet dairy DP were not significantly associated with the risk of lung cancer in moderate smokers. For moderate smokers, the risk of lung cancer was lower by 65% (OR: 0.35; 95% CI: 0.18–0.65; *p* < 0.05; adjusted model) and 66% (OR: 0.34; 95% CI: 0.15–0.76; *p* < 0.05; adjusted model) in the moderate adherence (4–6 points) and the high adherence (7–9 points) to the Polish-aMED score when compared to the low adherence (0–3 points).

No dietary pattern was significantly associated with the risk of lung cancer among heavy smokers.

## 4. Discussion

The current study found that men from northeastern Poland had higher adherence to the prudent DP and that the Polish-aMED score was associated with a significantly lower risk of lung cancer, independent of confounders. This inverse association between both pro-healthy dietary patterns and lung cancer in men was visible even in moderate smokers, although it was not confirmed among heavy smokers. The westernized traditional DP and sweet dairy DP were not significantly associated with the risk of lung cancer. 

The study showed that the risk of lung cancer was 28% lower in men with higher adherence to the prudent DP, independent of confounders, which were taking into consideration. This prudent DP included whole grain products, fruits, nuts and seeds, vegetables, fish, legumes, fruits, vegetables, and vegetable-fruit juices, while it was negatively loaded by the consumption frequency of refined grain products, sugar, honey, and sweets. A beneficial effect was previously shown for many pro-healthy dietary patterns, which were rich, similar to the prudent DP, in fruits and vegetables [29], milk [32], low-fat foods [14], fish [70], pasta, rice, poultry, and oils [31]. 

High and moderate adherence to the Polish-aMED score reduced lung cancer risk at the same level (by 49%), independent of confounders. This strong association was found even though Poland is a non-Mediterranean country and the dietary habits of the Polish population do not closely resemble the traditional Mediterranean diet. This result is consistent with previous outcomes from other countries, which found a beneficial effect of the Mediterranean pattern on lung cancer risk [44,68]. In a Dutch study (NLCS), high adherence to the alternate Mediterranean diet (without alcohol) was associated with a reduced risk of lung cancer in men by 9% and women by 27% [44]. In a study (both sexes) in Sweden, it was found that following the Mediterranean diet reduces the risk of lung cancer by 14%, while, in Italy, following the recommendations of this diet reduces the risk by as much as 80% [71,72]. While there were similarities between the countries, there are also important differences in dietary habits and food consumption [49]. An updated systematic review and meta-analysis confirm that high adherence to the Mediterranean diet reduces the risk of respiratory cancers by 16% [73]. 

An inverse association between lung cancer and both pro-healthy dietary patterns was visible even in moderate smokers. Moderate smokers with higher adherence to the prudent DP had a lower risk of lung cancer by 41%, independent of confounders. Previous studies from China [32], USA [29], and the Netherlands [31] revealed that the pro-healthy patterns rich in fruits and vegetables were associated with reduced risk of lung cancer risk among smokers. Case studies among smokers (both genders) found that the fruits and vegetables pattern was associated with the reduced risk of lung cancer by 46% in Americans and 73% in Chinese people [29,32]. For Dutch males, higher adherence to the salad vegetables pattern was associated with a 10% lower risk of lung cancer among male smokers and 23% lower risk among former smokers [31]. This result was partly confirmed in the current study, showing that for moderate smokers, a higher adherence to the prudent DP, characterized by a higher consumption frequency of vegetables and fruit, was associated with a lower risk of lung cancer.

The current study found that moderate smokers (including current smokers) with higher and moderate adherence to the Polish-aMED score had a lower risk of lung cancer (66% and 65%, respectively), independent of confounders, which were taken into consideration. Previous studies from Australia [45] found that high adherence to the Mediterranean diet score was associated with lower lung cancer risk (by 36% overall and by 62% for current smokers). The European Prospective Investigation into Cancer and Nutrition (EPIC) study [74] found that higher adherence to the Mediterranean dietary pattern was associated with a 12% reduction in the risk of tobacco-related cancers among smokers. Thus, the current results reinforce previous reports and confirm the pro-health effect of the Mediterranean diet in relation to the risk of lung cancer, even for moderate smokers.

The two pro-healthy dietary patterns considered in this study—the prudent pattern and the Polish a-MED score—were inversely associated with healthy outcomes in moderate smokers. Moderate male smokers with higher adherence to these dietary patterns had a lower risk of lung cancer (by 41% and 66%, respectively), whereas, among heavy male smokers, this relationship disappeared, and no pro-healthy dietary patterns were significantly associated with the risk of lung cancer. In contrast to the current findings, in an Italian study involving heavy smokers (≥20 pack-years), adherence to the vitamin and fiber diet regimen was associated with a 43% lower risk of lung cancer [75]. Although this pattern can be classified as pro-healthy, it was not similar to the prudent pattern in the current study because the vitamin and fiber formula included vitamin supplementation, so the possibility of comparing both results is limited. This finding documents the positive potential of pro-healthy dietary patterns. It may be speculated that these DPs could modulate the negative impact of moderate smoking, but not heavy smoking. Further studies are needed in both sexes and in other countries to determine if it is a general rule or a specific relationship for Polish males.

The study found no association between the westernized traditional pattern and lung cancer, which can be explained by the opposite influence of food components of this pattern. The westernized traditional pattern reflects the diet of many Poles, which consists of traditional staple foods (e.g., meat, potatoes, refined grain products) and vegetables prepared in a traditional manner, which are combined with western dietary influences, e.g., the consumption of sweetened beverages and energy drinks, sugar, honey, and sweets [76,77]. Contrary to the current results, a higher adherence to dietary patterns labelled as Western was associated with an increased risk of lung cancer in American [29] and Uruguayan men [36], although components of these Western patterns slightly differed from the westernized traditional pattern of Polish men. In previous Polish studies [33] regarding the risk of breast cancer in women and lung cancer in men, no significant relationship between the traditional Polish pattern and both cancers was obtained. The components of the westernized Polish pattern coincide with the characteristics reported in previous Polish studies, in which the westernized traditional pattern was positively associated with lipid disorders and high blood pressure in adolescents [76], and the westernized traditional pattern with premature coronary artery disease in young men [78].

In the current study, no association between the sweet dairy pattern and lung cancer risk was found. This pattern included foods with a potentially beneficial effect on health (vegetable oils, dried fruit, and preserves) and foods with potentially negative effects on health (sugar, honey, sweets, and breakfast cereals) [76,79,80]. Such a combination may have influenced the neutral character of the final results regarding lung cancer risk. Other studies on dietary patterns and lung cancer have not identified a similar pattern, so a direct comparison of the current results is limited. The components of the sweet dairy pattern coincide with the characteristics reported in previous health-related studies, in which the pattern was neutral for health outcomes. In elderly Italians from the EPIC study, more hyperlipidemic subjects were found in those with lower adherence to the sweet and dairy pattern than those with normal blood lipids [81].

In the current study, in moderate and heavy male smokers, the westernized traditional and the sweet dairy patterns were not significantly associated with the risk of lung cancer. No previous studies involved male smokers to investigate the risk of lung cancer in this cohort with similar dietary patterns, so a direct comparison of the current results with other studies cannot be provided.

To the authors’ best knowledge, this is the first Polish study in men regarding the association between lung cancer risk and dietary patterns, including the Polish-aMED score, considering the time of tobacco smoking. Overall, the data highlight the beneficial effect of a healthy diet on lung cancer risk in men from northeastern Poland. These findings could be helpful in preventing lung cancer, which was the most common cause of death from cancer in 14 regions of the world in 2018 (including in Eastern Europe) [82].

### Strength and Limitations

The current study has several limitations. Due to the multifactorial aetiology of cancer, an adjusted model for the diet–cancer association, including many potential confounders [7,33,34], was calculated. However, it was not possible to include all potential confounders. Thus, the possibility of residual confounding by factors that were not evaluated cannot be ruled out. The next limitation is a lack of quantitative data regarding food consumption and nutrient intake, although current evidence shows the limitations in concluding when single foods or nutrient components are considered [80]. In this study, an informational bias regarding lifestyle factors (e.g., diet, physical activity, smoking) is possible—it is a general limitation when subject-reported data are collected and interpreted. On the other hand, there is no golden method, and the food frequency method covering one year is a reasonable choice in dietary assessment [83]. Furthermore, the measurement of long-term usual daily physical activity or smoking is difficult. It cannot be ruled out that some of these lifestyle factors may have changed close to the cancer diagnosis due to early symptoms. Although retrospective data has been collected (for diet: 12 months before to data collection and diagnosis; for smoking: many years before). Thus, it can be speculated that the association between retrospective, long-time exposure (lifestyle) and cancer risk was identified, not short-term exposure as a result of health impairment. With respect to smoking, the cumulative exposure (in pack-years) can be interpreted with caution. Without a doubt, the longer one smokes and the more cigarettes one smokes per day (pack-years), the greater one’s chances of getting lung cancer and other smoking-related diseases. Even though the problem of how to summarize the effects of extended and variable exposures is a universal one in epidemiology and is still open, the cumulative dose has been widely used in analyses of numerous other exposures, including smoking [84]. In this study, a multidimensional statistical analysis (PCA) has both limitations and strengths. For the three PCA-driven dietary patterns that have been identified, the cumulative percentage of variance explained was not too high (31%). Since dietary data are variables with high variability [85], it is not possible to achieve a high level of the total variance explained while data-driven dietary patterns were identified. According to the literature on nutrition, the typical range is 20% to 30% of the total variance explained by two or three dietary factors (dietary patterns). In this light, the current result (31% for three factors) is better than many previously reported [86,87,88,89]. The strength of a statistical analysis is the sample size—sufficient for PCA performance (the subject-to-item ratio 19:1) [61].

A major strength of the study is the identification of dietary patterns (hypothesis-driven approach and data-driven approach) [58], which represents the overall combination of commonly consumed food and a consideration of the health outcome (synergistic or opposed) of many single dietary items [90]. Secondly, an additional logistic regression analysis was performed on the data, including an age-matched control sub-sample (*n* = 238), to ensure that study outcomes do not differ when the two groups are age-matched (Tables S6 and S7). Thirdly, to collect dietary data, an interviewer-administrated FFQ with adequate to high internal repeatability [91] was used. Finally, although several studies have explored the association between dietary patterns and lung cancer, none have reported results from adults living in Central or Eastern Europe, considering the history of tobacco smoking. The current paper fills this important gap by exploring the link between different dietary patterns, including the Polish-aMED score, and cancer risk in male smokers from northeastern Poland.

## 5. Conclusions

The current study suggests that pro-healthy dietary patterns, including the Mediterranean pattern, may favour a lower risk of lung cancer in men. This beneficial association was found in moderate smokers, although it was not confirmed in heavy smokers. It can be speculated that a higher consumption frequency of foods with components of a pro-healthy diet can, to some extent, reduce the negative effect of moderate smoking and lower the risk of lung cancer. Given the limited number of participants, prospective studies with close control of confounding factors are needed, especially in northeastern European countries, to confirm the link between pro-healthy dietary patterns and the lower risk of lung cancer in moderate smokers.

## Figures and Tables

**Table 1:** Characteristics of the lung cancer sample and control sample.

Variable	Cancer Control Sample		Cancer Sample		Control Sample		*p*-Value
	*n*	%	*n*	%	*n*	%	
**Sample size**	439	100.0	187	100.0	252	100.0	
Age (years *)	62.6 (7.2)		63.0 (7.1)		62.3 (7.3)		0.2586
45.0–49.9	12	2.8	3	1.6	9	3.6	<0.0001
50.0–54.9	41	9.3	12	6.4	29	11.5	
55.0–59.9	76	17.3	24	12.8	52	20.6	
60.0–64.9	133	30.3	51	27.3	82	32.5	
65.0–69.9	97	22.1	42	22.5	55	21.8	
70.0–74.9	47	10.7	29	15.5	18	7.1	
75.0–80.0	33	7.5	26	13.9	7	2.8	
BMI (kg/m^2^ *)	27.7 (4.8)		27.3 (4.9)		28.0 (4.7)		0.1354
<18.5	10	2.3	10	5.4	0	0.0	<0.0001
18.5–24.9	125	28.5	86	46.0	39	15.5	
25.0–29.9	160	36.4	55	29.4	105	41.7	
≥30	144	32.8	36	19.2	108	42.8	
Place of residence							
rural	152	34.6	67	35.8	85	33.7	0.5981
sub-urban	202	46.0	81	43.3	121	48.0	
urban	85	19.4	39	20.9	46	18.3	
Education level							
primary	104	23.7	72	38.5	32	12.7	<0.0001
secondary	258	58.8	104	55.6	154	61.1	
higher	77	17.5	11	5.9	66	26.2	
Economic situation							
below average	91	20.7	53	28.3	38	15.1	<0.0001
average	278	63.3	118	63.1	160	63.5	
above average	70	16.0	16	8.6	54	21.4	
Socioeconomic status (SES index) ^a^							
low	235	53.5	121	64.7	114	45.2	0.0002
average	87	19.,8	25	13.4	62	24.6	
high	117	26.6	41	21.9	76	30.2	
Physical activity at work ^b^							
low	223	50.8	115	61.5	108	42.9	0.0003
moderate	154	35.1	48	25.7	106	42.0	
high	62	14.1	24	12.8	38	15.1	
Physical activity in leisure time ^c^							
low	148	33.7	71	38.0	77	30.6	0.2570
moderate	236	53.8	95	50.8	141	55.9	
high	55	12.5	21	11.2	34	13.5	
Overall physical activity ^d^							
low	227	51.7	113	60.4	114	45.2	0.0069
moderate	165	37.6	57	30.5	108	42.9	
high	47	10.7	17	9.1	30	11.9	
Current smoking status							
never smokers	50	12.5	1	0.5	49	19.5	<0.0001
current smokers	157	34.6	69	36.9	88	45.6	
former smokers	232	52.9	117	62.6	115	34.9	
Smoking period							
never smokers	55	12.5	1	0.5	54	21.4	<0.0001
<5 years	27	6.1	5	2.7	22	8.7	
5–10 years	13	3.0	2	1.1	11	4.4	
>10 years	344	78.4	179	95.7	165	65.5	
Number of cigarettes smoked							
0 pcs	55	12.5	1	0.5	54	21.4	<0.0001
<10 pcs	28	6.4	6	3.2	22	8.7	
11–20 pcs	217	49.4	104	55.6	113	44.9	
21–40 pcs	104	23.7	50	26.8	54	21.4	
>40 pcs	35	8.0	26	13.9	9	3.6	
Smoking (pack-years)							
0 pack-years (never smoker)	55	12.5	1	0.5	54	21.4	<0.0001
2.5–11 pack-years (moderate smoker)	252	57.4	112	59.9	140	55.6	
>11 pack-years (heavy smoker)	132	30.1	74	39.6	58	23.0	
Family history of lung cancer among relatives							
yes	91	20.7	39	20.9	52	20.6	0.9945
no	324	73.8	138	73.8	186	73.8	
I do not know	28	5.5	10	5.3	14	5.6	
Occupational exposure in the workplace							
yes	137	31.2	64	34.2	73	28.9	0.2399
no	302	68.8	123	65.8	179	71.1	

* Mean and *standard deviation* (SD); ^a^ SES index was calculated based on place of residence, educational level, and declared economic situation (description in the Materials and Methods section); ^b^ physical activity at work: low—more than 70% of working time spent sedentary or retired, moderate—approx. 50% of working time spent sedentary and 50% of working time spent in an active manner, high—approx. 70% of working time spent in an active manner or physical work related to great exertion [64]; ^c^ physical activity in leisure time: low—sedentary for most of the time, watching TV, reading books, walking 1–2 h per week, moderate—walking, bike riding, gymnastics, gardening, light physical activity performed 2–3 h per week, high—bike riding, jogging, gardening, sport activities involving physical exertion performed more than 3 h weekly [68]; ^d^ after combining data based on declared physical activity at work and physical activity in leisure time [69]; details in Supplementary Table S3; *n*—number; %—percentage of the sample; *p*—level of significance assessed by chi^2^ test (categorical variables) or Kruskal–Wallis’ test (continuous variables).

**Table 2:** Factor loadings for food groups in principal component analysis (PCA)-derived dietary patterns and the Pearson’s correlation coefficients for food groups in the Polish-aMED score (*n* = 439).

Food Groups	PCA-Derived Dietary Patterns			Polish-aMED Score
	Prudent	Westernized Traditional	Sweet Dairy	
Whole grain products	**0.65**	−0.04	−0.03	0.49 *
Fruits	**0.64**	0.16	0.24	0.25 *
Nuts and seeds	**0.57**	0.05	0.12	0.54 *
Vegetables	**0.49**	**0.43**	0.20	0.50 *
Fish	**0.48**	0.23	0.08	**0.41**
Legumes	**0.47**	0.04	0.26	0.41 *
Fruit, vegetable, vegetable-fruit juices	**0.32**	0.20	0.26	0.21 *
Refined grains products	**−0.54**	**0.39**	**0.35**	−0.21 *
Sugar, honey, and sweets	**−0.45**	**0.37**	**0.37**	−0.09
Red and processed meats	0.08	**0.63**	0.07	**−0.00**
White meat	0.19	**0.56**	−0.01	0.13 *
Potatoes	−0.13	**0.51**	0.13	−0.02
Other fats	−0.18	**0.45**	**−0.39**	−0.13 *
Sweetened beverages and energy drinks	0.10	**0.38**	0.23	0.14 *
Animal fats	−0.11	0.01	**0.66**	0.06
Milk, fermented milk drinks, and curd cheese	0.13	0.12	**0.49**	0.17 *
Sweetened milk drinks and flavored homogenized cheese	−0.02	0.01	**0.45**	0.07
Eggs	0.12	0.17	**0.43**	0.14 *
Cheese	0.12	0.15	**0.38**	0.19 *
Breakfast cereals	0.10	−0.25	**0.37**	0.10 *
Vegetable oils	0.11	0.24	**0.33**	0.11 *
Dried fruit and preserves	0.23	0.02	**0.32**	0.15 *
Alcohol	0.05	0.12	0.01	0.21 *
Ratio of vegetable oils to animal fat	NA	NA	NA	0.11 *
Share in explaining the variance (%)	15	10	6	NA

Polish-aMED—Polish-adapted Mediterranean diet (range of points: 0–9); bolded values are marked for the main components of PCA-derived dietary patterns with absolute loadings > |0.3| and for nine components of the Polish-aMED score; * *p* < 0.05, test of significance for Pearson’s correlation coefficients; NA—not applied.

**Table 3:** Distribution of lung cancer cases by adherence to the dietary patterns and smoking status.

Adherence to the Dietary Pattern	Cancer Control Sample (*n* = 439)				Smoking Status											
					Never Smoker (*n* = 55)				Moderate Smoker (*n* = 252)				Heavy Smoker (*n* = 132)			
	Sample Size	Cancer Cases		*p*	Sample Size	Cancer Cases		*p*	Sample Size	Cancer Cases		*p*	Sample Size	Cancer Cases		*p*
		*n*	%			*n*	%			*n*	%			*n*	%	
Prudent				<0.0001				0.6742				0.0046				0.6541
lower	146	81	55.5		7	0	0.0		90	51	56.7		49	30	61.2	
moderate	146	64	43.8		17	0	0.0		85	41	48.3		44	23	52.3	
higher	147	42	28.6		30	1	3.3		77	20	25.9		40	21	52.5	
Westernized Traditional				0.8912				0.4102				0.9861				0.1221
lower	147	63	42.9		20	1	5.0		84	37	44.0		43	25	58.1	
moderate	146	64	43.8		19	0	0.0		82	37	45.1		45	27	60.0	
higher	146	60	41.1		16	0	0.0		86	38	44.2		44	22	50.0	
Sweet Dairy				0.4017				0.4101				0.9054				0.8343
lower	146	62	42.5		17	0	0.0		85	37	43.5		44	25	56.8	
moderate	147	57	38.8		20	1	5.0		89	37	41.6		38	19	50.0	
higher	146	68	46.6		18	0	0.0		78	38	48.7		50	30	60.0	
Polish–aMED score				<0.0001				0.4102				0.0003				0.8254
low (0–3 points)	177	97	51.9		15	0	0.0		106	63	59.4		56	34	60.7	
moderate (4–6 points)	221	83	44.4		27	1	3.7		127	47	37.1		67	35	52.2	
high (7–9 points)	41	7	3.7		13	0	0.0		19	2	10.5		9	5	55.6	

Polish-aMEDscore—Polish-adapted Mediterranean diet score (range of points: 0–9); *n*—number; %—percentage of the sample; *p*—the level of significance verified with the chi^2^ test (cancer cases vs. controls).

**Table 4:** Odds ratios (95% confidence intervals) of lung cancer risk by adherence to the dietary patterns.

Adherence to the Dietary Pattern	Cancer-Control Sample (*n* = 439)			
	Crude Model	*p*	Adjusted Model	*p*
Prudent				
lower (ref.)	Ref.		Ref.	
moderate	0.63 (0.39; 1.00)	0.0472	0.63 (0.37; 1.08)	0.1394
higher	0.57 (0.44; 0.72)	<0.0001	0.72 (0.53; 0.96)	0.0432
Westernized Traditional				
lower (ref.)	Ref.		Ref.	
moderate	1.04 (0.65; 1.66)	0.8667	0.79 (0.45; 1.37)	0.5123
higher	0.96 (0.76;1.22)	0.7601	0.81 (0.60; 1.08)	0.1495
Sweet Dairy				
lower (ref.)	Ref.		Ref.	
moderate	0.86 (0.54; 1.37)	0.5203	0.68 (0.39; 1.20)	0.1495
higher	1.09 (0.86; 1.37)	0.4803	0.99 (0.75; 1.30)	0.7999
Polish–aMED score				
low (0–3 points; ref.)	Ref.		Ref.	
moderate (4–6 points)	0.50 (0.33; 0.74)	0.0004	0.51 (0.31; 0.81)	0.0048
high (7–9 points)	0.41 (0.27; 0.64)	<0.0001	0.51 (0.32; 0.81)	0.0046

Ref.—reference category; Polish-aMEDscore—Polish-adapted Mediterranean diet score (range of points: 0–9); *p*—the level of significance assessed by Wald’s test; model adjusted for: age (categories), BMI (categories), current smoking status (never smokers, current smokers, former smokers), socioeconomic status (low, average, high), overall physical activity (low, moderate, high), the occurrence of lung cancer in relatives (yes, no, I do not know), and occupational exposure in the workplace (yes, no); reference category in the PCA: controls.

**Table 5:** Odds ratios (95% confidence intervals) of lung cancer risk by adherence to the dietary patterns in smokers.

Adherence to the Dietary Pattern	Moderate Smoker (*n* = 252)				Heavy Smoker (*n* = 132)			
	Crude Model	*p*	Adjusted Model	*p*	Crude Model	*p*	Adjusted Model	*p*
Prudent								
lower (ref.)	Ref.		Ref.		Ref.		Ref.	
moderate	0.67 (0.37; 1.21)	0.2579	0.68 (0.34; 1.34)	0.2579	0.80 (0.34; 1.90)	0.6226	0.82 (0.30; 2.26)	0.6958
higher	0.51 (0.37; 0.71)	< 0.0001	0.59 (0.39; 0.90)	0.0154	0.88 (0.57; 1.37)	0.5758	1.10 (0.64; 1.88)	0.7187
Westernized Traditional								
lower (ref.)	Ref.		Ref.		Ref.		Ref.	
moderate	1.09 (0.59; 2.00)	0.8320	0.92 (0.43; 1.96)	0.8320	0.95 (0.40; 2.33)	0.9147	0.79 (0.28; 2.27)	0.6574
higher	1.03 (0.76; 1.40)	0.8131	0.95 (0.65; 1.40)	0.8131	0.77 (050; 1.21)	0.2505	0.69 (0.40; 1.19)	0.1495
Sweet Dairy								
lower (ref.)	Ref.		Ref.		Ref.		Ref.	
moderate	0.96 (0.53; 1.76)	0.5139	0.78 (0.38; 1.64)	0.5139	0.68 (0.27; 1.69)	0.3944	0.49 (0.16; 1.46)	0.1496
higher	1.14 (0.84;1.56)	0.8109	1.05 (0.72; 1.51)	0.8109	0.98 (0.64; 1.50)	0.9244	0.92 (0.56; 1.50)	0.7999
Polish–aMED score								
low (0–3 points; ref.)	Ref.		Ref.		Ref.		Ref.	
moderate (4–6 points)	0.40 (0.23; 0.68)	0.0007	0.35 (0.18; 0.65)	0.0010	0.71 (0.34; 1.46)	0.3462	0.64 (0.27; 1.55)	0.2485
high (7–9 points)	0.28 (0.13; 0.61)	0.0010	0.34 (0.15; 0.76)	0.0079	0.77 (0.90; 0.44)	0.7692	1.17 (0.53; 2.61)	0.3462

Ref.—reference category; Polish-aMEDscore—Polish-adapted Mediterranean diet score (range of points: 0–9); moderate smoker—smoker who smoked 2.5 to 11 pack-years; heavy smoker—smoker who smoked more than 11 pack-years; *p*—the level of significance assessed by Wald’s test; adjusted model for: age (categories), BMI (categories), socioeconomic status (low, average, high), overall physical activity (low, moderate, high), the occurrence of lung cancer in relatives (yes, no, I do not know), and occupational exposure in the workplace (yes, no); reference category in the PCA: never smoker in controls.

## Supplementary Materials

The following are available online at https://www.mdpi.com/2072-6643/12/12/3788/s1, Table S1: Food groups used in the dietary pattern analysis. Table S2: The Polish-aMED score (0–9 points) calculation and reference medians of food consumption frequency—data for 262 subjects of the control sample. Table S3: Estimation of the overall physical activity after combining data based on self-reported physical activity at work and physical activity in leisure time. Table S4: The mean (95% confidence interval) of the frequency of food consumption by smoking status for the cancer control sample (times/day). Table S5: The mean (95% confidence interval) of the frequency of food consumption by dietary patterns (times/day). Table S6: Characteristics of the lung cancer sub-sample and the control sub-sample after age matching. Table S7: Odds ratios (95% confidence interval) of lung cancer risk by adherence to the dietary patterns in smokers after age matching of controls.

## References

[1] Bray F., Ferlay J., Soerjomataram I., Siegel R.L., Torre L.A., Jemal A. (2018). Global cancer statistics 2018: GLOBOCAN estimates of incidence and mortality worldwide for 36 cancers in 185 countries. CA Cancer J. Clin..

[2] Wild C.P., Weiderpass E., Stewart B.W. (2020). Editors 2020. World Cancer Report: Cancer Research for Cancer Prevention.

[3] Bade B.C., Dela Cruz C.S. (2020). Lung Cancer 2020: Epidemiology, Etiology, and Prevention. Clin. Chest Med..

[4] Wojciechowska U., Didkowska J. (2014). Zachorowania i zgony na Nowotwory Złośliwe w Polsce.

[5] OECD (2019). Health at a Glance 2019: OECD Indicators.

[6] Kathuria H., Neptune E. (2020). Primary and Secondary Prevention of Lung Cancer. Clin. Chest Med..

[7] World Cancer Research Fund/American Institute for Cancer Research Continuous Update Project Expert Report 2018. Diet, Nutrition, Physical Activity and Lung Cancer. https://www.wcrf.org/sites/default/files/Lung-cancer-report.pdf.

[8] Malhotra J., Malvezzi M., Negri E., La Vecchia C., Boffetta P. (2016). Risk factors for lung cancer worldwide. Eur. Respir. J..

[9] Bentley A.R., Kritchevsky S.B., Harris T.B., Holvoet P., Jensen R.L., Newman A.B., Lee J.S., Yende S., Bauer D. (2012). Health ABC Study. Dietary antioxidants and forced expiratory volume in 1 s decline: The Health, Aging and Body Composition study. Eur. Respir. J..

[10] Ng T.P., Niti M., Yap K.B., Tan W.C. (2014). Dietary and supplemental antioxidant and anti-inflammatory nutrient intakes and pulmonary function. Public Health Nutr..

[11] Garcia-Larsen V., Amigo H., Bustos P., Bakolis I., Rona R.J. (2015). Ventilatory function in young adults and dietary antioxidant intake. Nutrients.

[12] de Alencar V.T.L., Formiga M.N., de Lima V.C.C. (2020). Inherited lung cancer: A review. E Cancer Med. Sci..

[13] Sun S., Schiller J., Gazdar A. (2007). Lung cancer in never smokers—A different disease. Nat. Rev. Cancer.

[14] Gorlova O.Y., Weng S.F., Hernandez L., Spitz M.R., Forman M.R. (2011). Dietary patterns affect lung cancer risk in never smokers. Nutr. Cancer.

[15] Elisia I., Cho B., Hay M., Li M.Y., Hofs E., Lam V., Dyer R.A., Lum J., Krystal G. (2019). The effect of diet and exercise on tobacco carcinogen-induced lung cancer. Carcinogenesis.

[16] World Cancer Research Fund/American Institute for Cancer Research Diet, Nutrition, Physical Activity and Cancer: A Global Perspective. Continuous Update Project Expert Report 2018. https://www.wcrf.org/sites/default/files/Summary-of-Third-Expert-Report-2018.pdf.

[17] Vieira A.R., Abar L., Vingeliene S., Chan D.S., Aune D., Navarro-Rosenblatt D., Stevens C., Greenwood D., Norat T. (2016). Fruits, vegetables and lung cancer risk: A systematic review and meta-analysis. Ann. Oncol..

[18] Abar L., Vieira A.R., Aune D., Stevens C., Vingeliene S., Rosenblatt D.A.N., Chan D., Greenwood D.C., Norat T. (2016). Blood concentrations of carotenoids and retinol and lung cancer risk: An update of the WCRF-AICR systematic review of published prospective studies. Cancer Med..

[19] Lippi G., Mattiuzzi C., Cervellin G. (2016). Meat consumption and cancer risk: A critical review of published meta-analyses. Crit. Rev. Oncol. Hematol..

[20] Sharma P., McClees S.F., Afaq F. (2017). Pomegranate for prevention and treatment of cancer: An update. Molecules.

[21] Wakai K., Sugawara Y., Tsuji I., Tamakoshi A., Shimazu T., Matsuo K., Nagata C., Mizoue T., Tanaka K., Inoue M. (2015). Risk of lung cancer and consumption of vegetables and fruit in Japanese: A pooled analysis of cohort studies in Japan. Cancer Sci..

[22] Gilsing A.M., Weijenberg M.P., Goldbohm R.A., Dagnelie P.C., van den Brandt P.A., Schouten L.J. (2016). Vegetarianism, low meat consumption and the risk of lung, postmenopausal breast and prostate cancer in a population-based cohort study. Eur. J. Clin. Nutr..

[23] Luqman M., Javed M.M., Daud S., Raheem N., Ahmad J., Khan A.U. (2014). Risk factors for lung cancer in the Pakistani population. Asian Pac. J. Cancer Prev..

[24] Linseisen J., Rohrmann S., Bueno-de-Mesquita B., Büchner F.L., Boshuizen H.C., Agudo A., Gram I.T., Dahm C.C., Overvad K., Egeberg R. (2011). Consumption of meat and fish and risk of lung cancer: Results from the European Prospective Investigation into Cancer and Nutrition. Cancer Causes Control..

[25] Theodoratou E., Timofeeva M., Li X., Meng X., Ioannidis J.P.A. (2017). Nature, Nurture, and Cancer Risks: Genetic and Nutritional Contributions to Cancer. Annu. Rev. Nutr..

[26] Narita S., Saito E., Sawada N., Shimazu T., Yamaji T., Iwasaki M., Ishihara J., Takachi R., Shibuya K., Inoue M. (2018). Dietary consumption of antioxidant vitamins and subsequent lung cancer risk: The Japan Public Health center-based prospective study. Int. J. Cancer.

[27] Tucker K.L. (2010). Dietary patterns, approaches, and multicultural perspective. Appl. Physiol. Nutr. Metab..

[28] Reedy J., Wirfält E., Flood A., Mitrou P.N., Krebs-Smith S.M., Kipnis V., Midthune D., Leitzmann M., Hollenbeck A., Schatzkin A. (2010). Comparing 3 dietary pattern methods—Cluster analysis, factor analysis, and index analysis—With colorectal cancer risk: The NIH–AARP Diet and Health Study. Am. J. Epidemiol..

[29] Tu H., Heymach J.V., Wen C.P., Ye Y., Pierzynski J.A., Roth J.A., Wu X. (2016). Different dietary patterns and reduction of lung cancer risk: A large cases-control study in the U.S.. Sci. Rep..

[30] Sun Y., Li Z., Li J., Li Z., Han J. (2016). A Healthy Dietary Pattern Reduces Lung Cancer Risk: A Systematic Review and Meta-Analysis. Nutrients.

[31] Balder H.F., Goldbohm R.A., van den Brandt P.A. (2005). Dietary patterns associated with male lung cancer risk in the Netherlands Cohort Study. Cancer Epidemiol. Biomark. Prev..

[32] He F., Xiao R.D., Lin T., Xiong W.M., Xu Q.P., Li X., Liu Z.G., He B.C., Hu Z.J., Cai L. (2018). Dietary patterns, BCMO1 polymorphisms, and primary lung cancer risk in a Han Chinese population: A case-control study in Southeast China. BMC Cancer.

[33] Krusinska B., Hawrysz I., Slowinska M.A., Wadolowska L., Biernacki M., Czerwinska A., Golota J.J. (2017). Dietary patterns and breast or lung cancer risk: A pooled analysis of two case-control studies in northern-eastern Poland. Adv. Clin. Exp. Med..

[34] Krusinska B., Hawrysz I., Wadolowska L., Slowinska M.A., Biernacki M., Czerwinska A., Golota J.J. (2018). Associations of Mediterranean Diet and a Posteriori Derived Dietary Patterns with Breast and Lung Cancer Risk: A Case-Control Study. Nutrients.

[35] Anic G.M., Park Y., Subar A.F., Schap T.E., Reedy J. (2016). Index-based dietary patterns and risk of lung cancer in the NIH-AARP diet and health study. Eur. J. Clin. Nutr..

[36] De Stefani E., Boffetta P., Ronco A.L., Deneo-Pellegrini H., Acosta G., Gutiérrez L.P., Mendilaharsu M. (2008). Nutrient patterns and risk of lung cancer: A factor analysis in Uruguayan men. Lung Cancer.

[37] De Stefani E., Deneo-Pellegrini H., Boffetta P., Ronco A.L., Aune D., Acosta G., Mendilaharsu M., Brennan P., Ferro G. (2009). Dietary patterns and risk of cancer: A factor analysis in Uruguay. Int. J. Cancer.

[38] O’Sullivan A., Gibney M.J., Brennan L. (2011). Dietary intake patterns are reflected in metabolomics profiles: Potential role in dietary assessment studies. Am. J. Clin. Nutr..

[39] Schwingshackl L., Schwedhelm C., Galbete C., Hoffmann G. (2017). Adherence to Mediterranean Diet and Risk of Cancer: An Updated Systematic Review and Meta-Analysis. Nutrients.

[40] Schwingshackl L., Hoffmann G. (2014). Adherence to Mediterranean diet and risk of cancer: A systematic review and meta-analysis of observational studies. Int. J. Cancer.

[41] Schwingshackl L., Hoffmann G. (2015). Adherence to mediterranean diet and risk of cancer: An updated systematic review and meta-analysis of observational studies. Cancer Med..

[42] Sofi F., Macchi C., Abbate R., Gensini G.F., Casini A. (2014). Mediterranean diet and health status: An updated meta-analysis and a proposal for a literature-based adherence score. Public Health Nutr..

[43] Schwingshackl L., Hoffmann G. (2016). Does a Mediterranean-type diet reduce cancer risk?. Curr. Nutr. Rep..

[44] Schulpen M., van den Brandt P.A. (2018). Adherence to the Mediterranean diet and risk of lung cancer in the Netherlands Cohort Study. Br. J. Nutr..

[45] Hodge A.M., Bassett J.K., Shivappa N., Hebert J.R., English D.R., Giles G.G., Severi G. (2016). Dietary inflammatory index, Mediterranean diet score, and lung cancer: A prospective study. Cancer Causes Control.

[46] Gnagnarella P., Maisonneuve P., Bellomi M., Rampinelli C., Bertolotti R., Spaggiari L., Palli D., Veronesi G. (2013). Red meat, Mediterranean diet and lung cancer risk among heavy smokers in the COSMOS screening study. Ann. Oncol..

[47] Mentella M.C., Scaldaferri F., Ricci C., Gasbarrini A., Miggiano G.A.D. (2019). Cancer and Mediterranean diet: A review. Nutrients.

[48] Bosetti C., Gallus S., Trichopoulou A., Talamini R., Franceschi S., Nergi E., La Vecchia C. (2003). Influence of the Mediterranean diet on the risk of cancers of the upper aerodigestive tract. Cancer Epidemiol. Biomark. Prev..

[49] Noah A., Truswell A.S. (2001). There are many Mediterranean diets. Asia Pac. J. Clin. Nutr..

[50] Mohtadi K., Msaad R., Benalioua N., Jafri A., Meftah H., Elkardi Y., Lebrazi H., Kettani A., Derouiche A., Taki H. (2020). Sociodemographic and Lifestyle Factors Associated with Adherence to Mediterranean Diet in Representative Adult Population in Casablanca City, Morocco: A Cross-Sectional Study. J. Nutr. Metab..

[51] Castro-Barquero S., Lamuela-Raventós R., Doménech M., Estruch R. (2018). Relationship between polyphenol intake in the Mediterranean diet and obesity. Nutrients.

[52] Kwan H.Y., Chao X., Su T., Fu X., Tse A.K., Fong W.F., Yu Z.L. (2017). The anticancer and antiobesity effects of Mediterranean diet. Crit. Rev. Food Sci. Nutr..

[53] Siriwardhana N., Kalupahana N.S., Moustaid-Moussa N. (2012). Health benefits of n-3 polyunsaturated fatty acids: Eicosapentaenoic acid and docosahexaenoic acid. Adv. Food Nutr. Res..

[54] Lee J.T., Lai G.Y., Liao L.M., Subar A.F., Bertazzi P.A., Pesatori A.C., Freedman N.D., Landi M.T., Lam T.K. (2017). Nut Consumption and Lung Cancer Risk: Results from Two Large Observational Studies. Cancer Epidemiol. Biomark. Prev..

[55] Grosso G., Bella F., Godos J., Sciacca S., Del Rio D., Ray S., Galvano F., Giovannucci E.L. (2017). Possible role of diet in cancer: Systematic review and multiple meta-analyses of dietary patterns, lifestyle factors, and cancer risk. Nutr. Rev..

[56] Niedzwiedzka E., Wadolowska L., Kowalkowska J. (2019). Reproducibility of a Non-Quantitative Food Frequency Questionnaire (62-Item FFQ-6) and PCA-Driven Dietary Pattern Identification in 13–21-Year-Old Females. Nutrients.

[57] Lidia Wadolowska Website. http://www.uwm.edu.pl/edu/lidiawadolowska/.

[58] Previdelli Á.N., de Andrade S.C., Fisberg R.M., Marchioni D.M. (2016). Using two different approaches to assess dietary patterns: Hypothesis-driven and data-driven analysis. Nutrients.

[59] Kaiser H.F. (1974). An index of factorial simplicity. Psychometrika.

[60] Bartlett M.S. (1950). Test of significance in factor analysis. Br. J. Math Stat. Psychol..

[61] Osborne J.W., Costello A.B. (2004). Sample size and subject to item ratio in principal components analysis. Pract. Assess. Res. Eval..

[62] Field A. (2009). Discovering Statistics Using SPSS.

[63] Fung T.T., McCullough M.L., Newby P.K., Manson J.E., Meigs J.B., Rifai N., Willett W.C., Hu F.B. (2005). Diet-quality scores and plasma concentrations of markers of inflammation and endothelial dysfunction. Am. J. Clin. Nutr..

[64] NIAAA (2018). What Is a Standard Drink?. https://www.niaaa.nih.gov/alcohol-health/overview-alcohol-consumption/what-standard-drink.

[65] Barragán R., Coltell O., Asensio E.M., Francés F., Sorlí J.V., Estruch R., Salas-Huetos A., Ordovas J.M., Corella D. (2016). MicroRNAs and Drinking: Association between the Pre-miR-27a rs895819 Polymorphism and Alcohol Consumption in a Mediterranean Population. Int. J. Mol. Sci..

[66] Bernaards C.M., Twisk J.W., Snel J., Van Mechelen W., Kemper H.C. (2001). Is calculating pack-years retrospectively a valid method to estimate life-time tobacco smoking? A comparison between prospectively calculated pack-years and retrospectively calculated pack-years. Addiction.

[67] Armitage P., Berry G., Matthews J.N.S. (2001). Statistical Methods in Medical Research.

[68] Jarosz M., Taraszewska A. (2011). Nadwaga i otyłość oraz wybrane elementy stylu życia jako czynniki ryzyka GERD. (Overweight and obesity and selected lifestyle elements as risk factors for GERD). Postępy Nauk Med..

[69] Wadolowska L., Krusinska B., Gawecki J. (2014). The manual for developing nutritional data from the KomPAN questionnaire. Dietary Habits and Nutrition Beliefs Questionnaire and the Manual for Developing of Nutritional Data.

[70] De Stefani E., Ronco A.L., Deneo-Pellegrini H., Correa P., Boffetta P., Acosta G., Mendilaharsu M. (2011). Dietary patterns and risk of adenocarcinoma of the lung in males: A factor analysis in Uruguay. Nutr. Cancer.

[71] Bodén S., Myte R., Wennberg M., Harlid S., Johansson I., Shivappa N., Hébert J.R., Van Guelpen B., Nilsson L.M. (2019). The inflammatory potential of diet in determining cancer risk; A prospective investigation of two dietary pattern scores. PLoS ONE.

[72] Maisonneuve P., Shivappa N., Hébert R., Bellomi M., Rampinelli C., Bertolotti R., Spaggiari L., Palli D., Veronesi G., Gnagnarella P. (2016). Dietary inflammatory index and risk of lung cancer and other respiratory conditions among heavy smokers in the COSMOS screening study. Eur. J. Nutr..

[73] Morze J., Danielewicz A., Przybyłowicz K., Zeng H., Hoffmann G., Schwingshackl L. (2020). An updated systematic review and meta-analysis on adherence to mediterranean diet and risk of cancer. Eur. J. Nutr..

[74] Couto E., Boffetta P., Lagiou P., Ferrari P., Buckland G., Overvad K., Dahm C.C., Tjonneland A., O Olsen A., Clavelchapelon F. (2011). Mediterranean dietary pattern and cancer risk in the EPIC cohort. Br. J. Cancer.

[75] Gnagnarella P., Maisonneuve P., Bellomi M., Raffaella B., Bertolotti R., Spaggiari L., Palli D., Veronesi G. (2013). Nutrient intake and nutrient patterns and risk of lung cancer among heavy smokers: Results from the COSMOS screening study with annual low-dose CT. Eur. J. Epidemiol..

[76] Dlugosz A. (2017). Dietary Patterns, Adverse Health Outcomes, Socioeconomic Situation and Lifestyle of Adolescents from Less Urbanised Regions of Poland [Dissertations and Monographs].

[77] De Stefani E., Deneo-Pellegrini H., Mendilaharsu M., Ronco A., Carzoglio J.C. (1998). Dietary sugar and lung cancer: A case-control study in Uruguay. Nutr. Cancer.

[78] Osadnik T., Pawlas N., Lonnie M., Osadnik K., Lejawa M., Wadolowska L., Bujak K., Fronczek M., Reguła R., Gawlita M. (2018). Family History of Premature Coronary Artery Disease (P-CAD)-A Non-Modifiable Risk Factor? Dietary Patterns of Young Healthy Offspring of P-CAD Patients: A Case-Control Study (MAGNETIC Project). Nutrients.

[79] Ceci C., Lacal P.M., Tentori L., De Martino M.G., Miano R., Graziani G. (2018). Experimental Evidence of the Antitumor, Antimetastatic and Antiangiogenic Activity of Ellagic Acid. Nutrients.

[80] Hosseini M., Naghan P.A., Jafari A.M., Yousefifard M., Taslimi S., Khodadad K., Mohammadi F., Sadr M., Rezaei M., Mortaz E. (2014). Nutrition and lung cancer: A case control study in Iran. BMC Cancer.

[81] Pala V., Sieri S., Masala G., Palli D., Panico S., Vineis P., Sacerdote C., Mattiello A., Galasso R., Salvini S. (2006). Associations between dietary pattern and lifestyle, anthropometry and other health indicators in the elderly participants of the EPIC-Italy cohort. Nutr. Metab. Cardiovasc. Dis..

[82] Ferlay J., Colombet M., Soerjomataram I., Mathers C., Parkin D.M., Piñeros M., Znaor A., Bray F. (2019). Estimating the global cancer incidence and mortality in 2018: GLOBOCAN sources and methods. Int. J. Cancer.

[83] Food and Agriculture Organization (FAO) (2018). Dietary Assessment: A Resource Guide to Method Selection and Application in Low Resource Settings. Rome, Italy..

[84] Duncan C.T. (2014). Invited Commentary: Is It Time to Retire the “Pack-Years” Variable? Maybe Not!. Am. J. Epidemiol..

[85] Kant A.K. (2010). Dietary patterns: Biomarkers and chronic disease risk. Appl. Physiol. Nutr. Metab..

[86] Nguyen H.H., Wu F., Oddy W.H., Wills K., Winzenberg T., Jones G. (2020). Associations between dietary patterns and osteoporosis-related outcomes in older adults: A longitudinal study. Eur. J. Clin. Nutr..

[87] Ruano C., Henriquez P., Martínez-González M.Á., Bes-Rastrollo M., Ruiz-Canela M., Sanchez-Villegas A. (2013). Empirically derived dietary patterns and health-related quality of life in the SUN project. PLoS ONE.

[88] Trudeau K., Rousseau M.-C., Barul C., Csizmadi I., Parent M.-É. (2020). Dietary Patterns Are Associated with Risk of Prostate Cancer in a Population-Based Case-Control Study in Montreal, Canada. Nutrients.

[89] Williams B., Onsman A., Brown T. (2010). Exploratory factor analysis: A five-step guide for novices. J. Emerg. Prim. Health Care.

[90] Wirfält E., Drake I., Wallström P. (2013). What do review papers conclude about food and dietary patterns?. Food Nutr. Res..

[91] Kowalkowska J., Wadolowska L., Czarnocinska J., Czlapka-Matyasik M., Galinski G., Jezewska-Zychowicz M., Bronkowska M., Długosz A., Loboda D., Wyka J. (2018). Reproducibility of a Questionnaire for Dietary Habits, Lifestyle and Nutrition Knowledge Assessment (KomPAN) in Polish Adolescents and Adults. Nutrients.

