# Supplementary material for: Adherence to Prudent and Mediterranean Dietary Patterns Is Inversely Associated with Lung Cancer in Moderate But Not Heavy Male Polish Smokers: A Case-Control Study
Source: Nutrients. 2020 Dec 10;12(12):3788. doi: 10.3390/nu12123788 (PMC7764397; doi:10.3390/nu12123788)
Supplement: Supplementary file 1 [file nutrients-12-03788-s001.pdf]

Table S1. Food groups used in the dietary pattern analysis.

Food Groups	Dietary Items
Whole grain products	Wholemeal wheat or rye bread, seeded loaves, pumpernickel, wholemeal cracker bread, buckwheat groats, barley, brown rice, wholemeal pasta, etc.
Fruits	All kinds of fruits
Nuts and seeds	Peanuts, hazelnuts, walnuts, cashews, coconuts, chestnuts, peanut butter, chocolate-nut spread, pumpkin seeds, sesame seeds, sunflower seeds, wheat germs, wheat bran, etc.
Vegetables	All kinds of vegetables (potatoes not included)
Fish	Pollock, cod, perch, hake, carp to 1 kg, tuna, panga, trout, salmon, sardines, herring, mackerel, eel, large carp, etc.
Legumes	Corn, green peas, green beans, etc.
Fruit, vegetable, vegetable-fruit juices	Mixed fruit juice, orange, grapefruit, apple, pear, grape, blackcurrant, cherry juice, mixed vegetable juice, tomato, carrot and carrot-fruit juice, etc.
Refined grain products	White bread, rye, wheat-rye bread, toast bread, white bread rolls, brioche, bagels, semolina, milled barley, pasta, white rice, rice flakes, etc.
Sugar, honey and sweets	Sugar added to beverages, such as tea, coffee. Honey added to dishes and beverages. Chocolate, chocolate sweets and chocolate bars, boiled sweets, hard caramels, jellied sweets, fudge, biscuits, cream cakes, fruit cakes, sponge cakes, cheesecakes, doughnuts, poppy-seed cakes, muffins, croissants, ice-creams and custard etc.
Red and processed meats	Pork, beef, veal, wild boar, venison, quail, mallard, hare, etc.
White meat	Poultry and rabbit
Potatoes	Boiled, baked, French fries, potato rosti, gnocchi, etc.
Other fats	Margarine for baking, frying, spreading. Mayonnaise and salad dressings
Sweetened beverages and energy drinks	Sweetened beverages and energy drinks
Animal fats	Lard, pork fat, etc.
Milk, fermented milk drinks and curd cheese	Milk and natural milk beverages (yoghurt, kefir, buttermilk), porridge, cheese curd, natural cottage cheese, soft cheese, mozzarella, cottage cheese with herbs, etc.
Sweetened milk drinks and flavoured homogenized cheese	Fruit yoghurts, yoghurts with chocolate flakes, flavoured buttermilk, hot chocolate, flavoured curds (with fruit, chocolate, vanilla), etc.
Eggs	Scrambled eggs, omelette, egg salad, cooked eggs
Cheese	Hard cheese, blue cheese, processed cheese, cheese spreads, etc.
Breakfast cereals	Muesli, cornflakes, other cereals - sweetened or unsweetened, etc.
Vegetable oils	Vegetable-based oil
Dried fruit, fruit preserves and fruit condiments	Raisins, dried apricots, figs, apples, prunes, fruit compote, jams, marmalades, dates, dried bananas, etc.
Alcohol	Beer, wine and cocktails, vodka and other spirits

Table S2. The 'Polish-aMED' score (0–9 points) calculation and reference medians of food consumption frequency–data for 262 subjects of the control sample.

Food Groups	Frequency of consumption (times/day)*		Criteria for 1 point
	**Mean (95% Cl)**	**Median#**	
Vegetables	0.831 (0.758; 0.903)	0.704	Greater than median intake
Fruit	0.625 (0.583; 0.666)	0.565	Greater than median intake
Whole grains	0.711 (0.633; 0.789)	0.671	Greater than median intake
Fish	0.233 (0.198; 0.268)	0.200	Greater than median intake
Legumes	0.207 (0.171; 0.244)	0.125	Greater than median intake
Nuts and seeds	0.258 (0.201; 0.316)	0.050	Greater than median intake
Ratio of vegetable oils to animal fat	1.334 (0.824; 1.844)	0.444	Greater than median intake
Red and processed meat	1.998 (1.856; 2.214)	1.913	Less than median intake
Alcohol	-	-	Do not abuse&

* food consumption frequency was expressed as times/day after assigning the values for categories of frequencies as follows: 'never or almost never' = 0; 'once a month or less' = 0.025; 'several times a month' = 0.1; 'several times a week' = 0.571; 'daily' = 1; 'several times a day' = 2. 95% CI—95% confidence interval; # reference median of food consumption frequency; & the men were asked about their alcohol consumption and, based on the literature, two classifications were created: (i) abuse, (ii) do not abuse alcohol [63, 64, 65].

Table S3. Estimation of the overall physical activity after combining data based on self-reported physical activity at work and physical activity in leisure time [69].

		**Physical activity at work**		
		**low**	**moderate**	**high**
**Physical activity in leisure time**	**low**	low	low	moderate
	**moderate**	low	moderate	moderate
	**high**	moderate	moderate	high

Table S4. The mean (95% Confidence Interval) of the frequency of food consumption by smoking status* for cancer-control sample (times/day).

Food groups	Moderate smoker			Heavy smoker		
	Cancer Sample	Control Sample	*p*	Cancer Sample	Control Sample	*p*
Sample size (n)	112	140	-	74	58	-
Sugar, honey and sweets	2.0 (1.9; 2.0)	1.9 (1.7; 2.0)	0.012	2.0 (1.9; 2.0)	2.0 (1.7; 2.0)	0.910
Red and processed meats	1.9 (1.7; 2.0)	2.0 (1.8; 1.9)	0.130	1.6 (1.4; 1.9)	2.0 (1.7; 2.2)	0.044
Animal fats	1.4 (1.2; 1.5)	1.1 (1.0; 1.3)	0.071	1.4 (1.2; 1.6)	1.3 (1.0; 1.5)	0.447
Refined grain products	1.3 (1.2; 1.5)	1.0 (0.9; 1.1)	0.001	1.3 (1.8; 1.4)	1.2 (1.0; 1.4)	0.508
Milk, fermented milk drinks and curd cheese	0.9 (0.7; 0.9)	1.0 (0.9; 1.1)	0.113	1.0 (0.8; 1.1)	1.0 (0.8; 1.2)	0.532
Vegetables	0.5 (0.4; 0.5)	0.6 (0.6; 0.7)	< 0.001	0.5 (0.4; 0.6)	0.5 (0.4; 0.6)	0.733
Fruits	0.6 (0.5; 0.6)	0.7 (0.6; 0.8)	0.024	0.6 (0.5; 0.7)	0.6 (0.5; 0.7)	0.846
Potatoes	0.6 (0.6; 0.7)	0.6 (0.6; 0.7)	0.998	0.6 (0.6; 0.7)	0.8 (0.7; 0.9)	0.009
Whole grain products	0.4 (0.3; 0.5)	0.7 (0.6; 0.8)	< 0.001	0.5 (0.4; 0.6)	0.6 (0.4; 0.7)	0.378
Vegetable oils (including olive oil)	0.5 (0.4; 0.6)	0.5 (0.5; 0.6)	0.238	0.5 (0.4; 0.5)	0.5 (0.4; 0.6)	0.414
Other fats (margarine, mayonnaise, dressings)	0.4 (0.3; 0.5)	0.4 (0.3; 0.5)	0.657	0.4 (0.2; 0.5)	0.5 (0.3; 0.6)	0.211
Cheese	0.4 (0.3; 0.5)	0.4 (0.4; 0.5)	0.582	0.4 (0.3; 0.4)	0.3 (0.2; 0.4)	0.566
White meat	0.4 (0.3; 0.4)	0.4 (0.4; 0.5)	0.068	0.3 (0.3; 0.4)	0.4 (0.4; 0.5)	0.086
Sweetened beverages and energy drinks	0.4 (0.3; 0.5)	0.4 (0.3; 0.5)	0.838	0.4 (0.3; 0.5)	0.3 (0.2; 0.4)	0.036
Dried fruit and preserves	0.1 (0.1; 0.1)	0.1 (0.1; 0.2)	0.295	0.1 (0.1; 0.1)	0.1 (0.1; 0.2)	0.146
Eggs	0.3 (0.2; 0.3)	0.4 (0.3; 0.4)	0.022	0.3 (0.3; 0.4)	0.4 (0.3; 0.4)	0.366
Alcohol	0.4 (0.3; 0.5)	0.4 (0.3; 0.5)	0.610	0.2 (0.1; 0.3)	0.2 (0.1; 0.4)	0.981
Sweetened milk drinks and flavoured homogenized cheese	0.2 (0.1; 0.2)	0.1 (0.1; 0.2)	0.476	0.2 (0.1; 0.3)	0.2 (0.1; 0.2)	0.168
Fish	0.2 (0.1; 0.2)	0.2 (0.2; 0.3)	0.229	0.2 (0.1; 0.3)	0.2 (0.1; 0.3)	0.915
Nuts and seeds	0.1 (0.0; 0.1)	0.3 (0.2; 0.4)	< 0.001	0.1 (0.0; 0.2)	0.1 (0.1; 0.2)	0.504
Legumes	0.1 (0.1; 0.1)	0.2 (0.2; 0.3)	0.002	0.2 (0.1; 0.3)	0.2 (0.1; 0.2)	0.705
Fruit, vegetable, vegetable-fruit juices	0.2 (0.1; 0.2)	0.1 (0.1; 0.2)	0.290	0.1 (0.1; 0.2)	0.1 (0.0; 0.2)	0.396
Breakfast cereals	0.0 (0.0; 0.1)	0.1 (0.0; 0.1)	0.287	0.0 (0.0; 0.1)	0.0 (0.0; 0.1)	0.545

* Data for never smokers were not presented due to the small number of subjects (n = 55) especially within the cancer sample (n = 1); *p* – level of significance verified with a Kruskal-Wallis test.

Table S5. The mean (95% Confidence Interval) of the frequency of food consumption by dietary patterns (times/day).

Food groups	Total	Adherence to the dietary pattern											
		'Prudent'			'Westernized Traditional'			'Sweet Dairy'			'Polish-a MED' score		
		lower	moderate	higher	lower	moderate	higher	lower	moderate	higher	low	moderate	high
Sample size (n)	439	146	146	147	147	146	146	146	147	146	177	221	41
Sugar, honey and sweets	2.0 (1.9;2.2)	3.0 (2.8;3.1)	2.0 (1.8;2.1)	1.2 (1.0;1.4)	1.2 (1.1;1.4)	2.0 (1.8;2.1)	2.9 (2.7;3.1)	1.3 (1.1;1.4)	2.0 (1.7;2.1)	2.9 (2.7;3.1)	2.4 (1.1;1.4)	2.0 (1.2;1.4)	1.0 (0.8;1.3)
Red and processed meats	1.9 (1.8;2.0)	1.9 (1.8;2.1)	1.9 (1.7;2.1)	2.0 (1.8;2.2)	1.1 (1.0;1.2)	1.9 (1.7;2.0)	2.9 (2.7;3.1)	1.6 (1.4;1.7)	1.9 (1.7;2.1)	2.4 (2.2;2.6)	1.8 (1.0;1.2)	2.1 (1.1;1.3)	2.1 (0.9;1.5)
Animal fats	1.3 (1.2;1.3)	1.4 (1.2;1.5)	1.3 (1.1;1.4)	1.2 (1.0;1.2)	1.1 (1.0;1.2)	1.2 (1.1;1.3)	1.5 (1.4;1.7)	0.6 (0.5;0.6)	1.2 (1.1;1.3)	2.0 (1.9;2.1)	1.3 (0.8;1.0)	1.3 (0.8;1.0)	0.8 (0.5;0.8)
Refined grain products	1.1 (1.0;1.2)	1.8 (1.7;1.8)	1.1 (1.0;1.2)	0.5 (0.5;0.6)	0.7 (0.6;08)	1.1 (1.0;1.2)	1.6 (1.5;1.7)	0.8 (0.7;0.9)	1.0 (0.9;1.1)	1.6 (1.5;1.7)	1.3 (0.7;0.9)	1.1 (0.7;0.8)	0.7 (0.5;0.7)
Milk, fermented milk drinks and curd cheese	0.9 (0.9;1.0)	0.9 (0.7;1.0)	0.9 (0.8;1.0)	1.1 (1.0;1.2)	0.8 (0.7;0.9)	0.9 (0.8;1.0)	1.1 (1.0;1.2)	0.6 (0.5;0.6)	1.0 (0.9;1.1)	1.2 (1.1;1.4)	0.8 (0.6;0.7)	1.0 (0.6;0.7)	1.0 (0.5;0.8)
Vegetables	0.8 (0.8;0.9)	0.8 (0.7;0.9)	0.8 (0.7;0.8)	0.9 (0.8;1.0)	0.7 (0.6;0.7)	0.8 (0.7;0.9)	1.0 (0.9;1.1)	0.7 (0.6;0.7)	0.8 (0.7;0.9)	1.0 (0.9;1.1)	0.7 (0.4;0.5)	0.9 (0.4:0.5)	1.0 (0.3;0.5)
Fruits	0.8 (0.7;0.8)	0.6 (0.6;0.7)	0.7 (0.7;0.8)	0.9 (0.8;1.0)	0.6 (0.6;0.7)	0.7 (0.6;0.8)	0.9 (0.9;1.0)	0.6 (0.5;0.7)	0.8 (0.7;0.8)	0.9 (0.9;1.0)	0.5 (0.4;0.5)	0.9 (0.4;0.5)	1.2 (0.4;0.6)
Potatoes	0.7 (0.6;0.7)	0.7 (0.7;0.7)	0.7 (0.6;0.7)	0.6 (0.5;0.7)	0.5 (0.4;0.6)	0.7 (0.7;0.8)	0.8 (0.7;0.8)	0.6 (0.5;0.6)	0.7 (0.6;0.7)	0.7 (0.7;0.8)	0.7 (0.3;0.4)	0.7 (0.3;0.4)	0.6 (0.2;0.4)
Whole grain products	0.6 (0.5;0.6)	0.2 (0.1;0.2)	0.5 (0.4;0.5)	1.2 (1.1;1.2)	0.7 (0.6;0.8)	0.6 (0.5;0.7)	0.5 (0.4;0.6)	0.6 (0.5;0.7)	0.7 (0.6;0.7)	0.5 (0.4;0.6)	0.3 (0.4;0.5)	0.7 (0.6;0.7)	1.2 (0.4;0.7)
Vegetable oils (including olive oil)	0.5 (0.5;0.5)	0.5 (0.4;0.5)	0.5 (0.4;0.5)	0.6 (0.5;0.6)	0.4 (0.3;0.5)	0.5 (0.5;0.6)	0.5 (0.5;0.6)	0.4 (0.3;0.4)	0.5 (0.5;0.6)	0.6 (0.6;0.7)	0.4 (0.3;0.4)	0.5 (0.3;0.4)	0.6 (0.3;0.4)
Other fats (margarine, mayonnaise, dressings)	0.4 (0.3;0.4)	0.5 (0.4;0.6)	0.4 (0.3;0.5)	0.2 (0.2;0.3)	0.2 (0.1;0.2)	0.4 (0.3;0.5)	0.6 (0.4;0.7)	0.6 (0.5;0.7)	0.4 (0.3;0.5)	0.2 (0.1;0.3)	0.4 (0.6;0.7)	0.4 (0.5;0.6)	0.4 (0.5;0.7)
Cheese	0.4 (0.4;0.4)	0.3 (0.3;0.4)	0.4 (0.3;0.5)	0.4 (0.4;0.5)	0.3 (0.3;0.4)	0.4 (0.3;0.4)	0.5 (0.4;0.5)	0.2 (0.2;0.3)	0.4 (0.4;0.5)	0.5 (0.5;0.6)	0.3 (0.3;0.3)	0.4 (0.2;0.3)	0.4 (0.3;0.4)
White meat	0.4 (0.4;04)	0.3 (0.3;0.4)	0.4 (0.4;0.4)	0.4 (0.4;0.5)	0.3 (0.2;0.3)	0.4 (0.4;0.4)	0.5 (0.5;0.5)	0.3 (0.3;0.3)	0.4 (0.4;0.5)	0.4 (0.4;0.5)	0.3 (0.4;0.3)	0.4 (0.2;0.3)	0.5 (0.2;0.3)
Sweetened beverages and energy drinks	0.4 (0.3;0.4)	0.4 (0.3;0.4)	0.4 (0.3;0.5)	0.4 (0.3;0.5)	0.2 (0.2;0.3)	0.3 (0.3;0.4)	0.6 (0.5;0.7)	0.3 (0.2;0.3)	0.3 (0.3;0.4)	0.6 (0.5;0.6)	0.3 (0.4;0.5)	0.4 (0.4;0.5)	0.4 (0.4;0.7)
Dried fruit and preserves	0.3 (0.4;0.4)	0.2 (0.3;0.4)	0.3 (0.3;0.4)	0.3 (0.4;0.5)	0.3 (0.4;0.5)	0.3 (0.3;0.4)	0.3 (0.3;0.4)	0.2 (0.3;0.4)	0.3 (0.4;0.5)	0.3 (0.4;0.4)	0.2 (0.3;0.4)	0.3 (0.3;0.4)	0.4 (0.4;0.6)
Eggs	0.3 (0.3;0.3)	0.3 (0.2;0.3)	0.3 (0.3;0.3)	0.4 (0.3;0.4)	0.2 (0.2;0.3)	0.3 (0.3;0.4)	0.4 (0.3;0.4)	0.2 (0.2;0.3)	0.3 (0.3;0.4)	0.4 (0.4;0.5)	0.2 (0.2;0.2)	0.3 (0.3;0.3)	0.4 (0.2;0.3)
Alcohol	0.3 (0.3;0.4)	0.4 (0.3;0.5)	0.2 (0.2;0.3)	0.4 (0.3;0.5)	0.2 (0.2;0.2)	0.4 (0.3;0.5)	0.4 (0.3;0.5)	0.3 (0.2;0.4)	0.4 (0.3;0.4)	0.3 (0.3;0.4)	0.4 (0.3;0.4)	0.3 (0.3;0.4)	0.3 (0.1;0.4)
Sweetened milk drinks and flavoured homogenized cheese	0.2 (0.1;0.2)	0.2 (0.1;0.2)	0.1 (0.1;0.2)	0.2 (0.1;0.2)	0.1 (0.1;0.1)	0.2 (0.1;0.2)	0.2 (0.2;0.3)	0.1 (0.0;0.1)	0.2 (0.1;0.2)	0.3 (0.2;0.3)	0.1 (0.2;0.3)	0.2 (0.3;0.4)	0.1 (0.2;0.3)
Fish	0.2 (0.2;0.2)	0.1 (0.1;0.2)	0.2 (0.2;0.2)	0.3 (0.3;0.4)	0.2 (0.1;0.2)	0.2 (0.2;0.3)	0.3 (0.2;0.3)	0.2 (0.1;0.2)	0.2 (0.2;0.3)	0.2 (0.2;0.3)	0.1 (0.1;0.1)	0.2 (0.2;0.3)	0.5 (0.4;0.6)
Nuts and seeds	0.2 (0.1;0.2)	0.1 (0.1;0.1)	0.1 (0.1;0.1)	0.4 (0.3;0.5)	0.2 (0.1;0.2)	0.2 (0.1;0.2)	0.2 (0.1;0.3)	0.2 (0.1:0.2)	0.2 (0.1;0.2)	0.3 (0.2;0.3)	0.1 (0.2;0.2)	0.2 (0.4;0.5)	0.6 (0.5;0.8)
Legumes	0.2 (0.1;0.2)	0.1 (0.1;0.2)	0.1 (0.1;0.2)	0.3 (0.2;0.3)	0.2 (0.1;0.2)	0.2 (0.1;0.2)	0.2 (0.2;0.3)	0.1 (0.1;0.2)	0.2 (0.1;0.2)	0.2 (0.2;0.3)	0.1 (0.1;0.2)	0.2 (0.2;0.3)	0.4 (0.3;0.5)
Fruit, vegetable, vegetable-fruit juices	0.1 (0.1;0.2)	0.1 (0.1;01)	0.1 (0.1;0.2)	0.2 (0.1;0.2)	0.1 (0.1;0.1)	0.1 (0.1;0.2)	0.2 (0.1;0.2)	0.1 (0.1;0.1)	0.1 (0.1;0.2)	0.2 (0.1;0.2)	0.1 (0.2;0.2)	0.1 (0.2;0.3)	0.3 (0.3;0.4)
Breakfast cereals	0.0 (0.0;0.1)	0.0 (0.0;0.0)	0.0 (0.0;0.0)	0.7 (0.0;0.1)	0.1 (0.0;0.1)	0.0 (0.0;0.0)	0.0 (0.0;0.1)	0.0 (0.0;0.0)	0.0 (0.0;0.1)	0.1 (0.0;0.1)	0.0 (0.1;0.1)	0.0 (0.1;0.2)	0.1 (0.2;0.2)

Table S6. Characteristics of the lung cancer sub-sample and the control sub-sample after age matching.

Variable	Cancer-control sample		Cancer sample		Control sub-sample		*p*
	n	%	n	%	n	%	
Sample size	406	100.0	168	100.0	238	100.0	
Age (years)*	62.3 (6.4)		63.4 (6.7)		61.6 (6.2)		0.5580
45.0-49.9	7	1.7	3	1.8	4	1.7	
50.0-54.9	37	9.1	12	7.1	25	10.5	
55.0-59.9	72	17.8	24	14.3	48	20.2	
60.0-64.9	132	32.5	51	30.3	81	34.0	0.0590
65.0-69.9	97	23.9	42	25.0	55	23.1	
70.0-74.9	47	11.6	29	17.3	18	7.6	
75.0-80.0	14	3.4	7	4.2	7	2.9	

*mean and standard deviation (SD); n—number; %—percentage of the sample; *p*—level of significance assessed by chi^2 test

Table S7. Odds ratios (95% Confidence Interval) of lung cancer risk by adherence to the dietary patterns in ever smokers after age matching of controls.

Adherence to the dietary pattern	Cancer-control sample (n=406)				Moderate smoker (n=168)				Heavy smoker (n=238)			
	**Crude model**	***p***	**Adjusted model**	***p***	**Crude model**	***p***	**Adjusted model**	***p***	**Crude model**	***p***	**Adjusted model**	***p***
**'Prudent'**												
lower (ref.)	Ref.		Ref.		Ref.		Ref.		Ref.		Ref.	
moderate	0.61 (0.37; 0.99)	0.0438	0.71 (0.41; 1.21)	0.2044	0.60 (0.33; 1.12)	0.1064	0.73 (0.37; 1.45)	0.3732	0.84 (0.34; 2.07)	0.7004	0.80 (0.88; 2.24)	0.6662
higher	0.54 (0.42; 0.70)	<0.0001	0.74 (0.55; 0.99)	0.0411	0.46 (0.33; 0.66)	<0.0001	0.64 (0.42; 0.96)	0.0313	0.62 (0.89; 0.57)	0.6244	1.04 (0.60; 1.80)	0.8823
**'Westernized Traditional'**												
lower (ref.)	Ref.		Ref.		Ref.		Ref.		Ref.		Ref.	
moderate	1.06 (0.66; 1.72)	0.0518	0.82 (0.47; 1.41)	0.4648	1.15 (0.61; 2.19)	0.6649	0.84 (0.40; 1.77)	0.6485	0.92 (0.37; 2.29)	0.8570	0.91 (0.33; 2.52)	0.8581
higher	0.98 (0.77; 1.26)	0.9034	0.83 (0.62; 1.11)	0.1999	1.07 (0.78; 1.47)	0.6625	0.94 (0.63; 1.39)	0.7514	0.81 (0.51; 1.28)	0.3571	0.75 (0.44; 1.28)	0.2899
**'Sweet Dairy'**												
lower (ref.)	Ref.		Ref.		Ref.		Ref.		Ref.		Ref.	
moderate	0.85 (0.52; 1.39)	0.5161	0.79 (0.46; 1.36)	0.3966	0.88 (0.47; 1.66)	0.7031	0.87 (0.43; 1.76)	0.6876	0.77 (0.30; 2.01)	0.5903	0.66 (0.22; 1.98)	0.4504
higher	1.09 (0.86; 1.39)	0.4631	1.02 (0.78; 1.34)	0.8444	1.12 (0.82; 1.55)	0.4641	0.98 (0.68; 1.43)	0.9317	1.02 (0.66; 1.58)	0.0056	1.06 (0.65; 1.73)	0.8068
**'Polish–aMED' score**												
low (0-3 points; ref.)	Ref.		Ref.		Ref.		Ref.		Ref.		Ref.	
moderate (4-6 points)	0.45 (0.29; 0.69)	0.0002	0.53 (0.33; 0.83)	0.0062	0.33 (0.19; 0.57)	<0.0001	0.35 (0.19; 0.66)	0.0009	0.68 (0.31; 1.50)	0.3376	0.70 (0.29; 1.68)	0.4139
high (7-9 points)	0.41 (0.27; 0.64)	<0.0001	0.52 (0.32; 0.82)	0.0053	0.28 (0.13; 0.61)	0.0012	0.36 (0.16; 0.80)	0.0109	0.88 (0.42; 1.84)	0.7298	1.15 (0.50; 2.62)	0.7347

Ref.—reference category; 'Polish-aMED'score—'Polish-adapted Mediterranean Diet' score (range of points: 0–9); Moderate smoker—smoker who smoked 2.5 to 11 pack-years; Heavy smoker—smoker who smoked more than 11 pack-years; *p*—the level of significance was assessed by Wald's test; Adjusted model for: status smoking currently only cancer-control sample (never smokers, current smokers, former smokers), BMI (categories), socioeconomic status (low, average, high), overall physical activity (low, moderate, high), the occurrence of lung cancer in relatives (yes, no, I do not know), occupational exposure in the workplace (yes, no); reference category in the PCA: never smoker in controls.
